# An interdisciplinary consensus on the management of bone metastases from renal cell carcinoma

**DOI:** 10.1038/s41585-018-0034-9

**Published:** 2018-06-14

**Authors:** Viktor Grünwald, Berit Eberhardt, Axel Bex, Anne Flörcken, Thomas Gauler, Thorsten Derlin, Martin Panzica, Hans Roland Dürr, Knut Achim Grötz, Rachel H. Giles, Christian von Falck, Anno Graser, Alexander Muacevic, Michael Staehler

**Affiliations:** 1 Department of Hematology, Hemostasis, Oncology and Stem Cell Transplantation, Hannover Medical School, Hannover, Germany; 2 Uronauten e.V., Berlin, Germany; 3 International Kidney Cancer Coalition, Duivendrecht, Netherlands; 4 Division of Surgical Oncology, Department of Urology, The Netherlands Cancer Institute, Amsterdam, Netherlands; 5 Department of Hematology, Oncology, and Tumor Immunology, Charité University Medicine Berlin, Berlin, Germany; 6 Westdeutsches Tumorzentrum, Universitätsklinik Essen, Essen, Germany; 7 Department of Nuclear Medicine, Hannover Medical School, Hannover, Germany; 8 Clinic of Trauma Surgery, Medical School Hannover, Hannover, Germany; 9 Orthopaedic Oncology, Department of Orthopaedic Surgery, Ludwig-Maximilians University Munich, Munich, Germany; 10 Department of Oral and Maxillofacial Surgery of the Dr Horst Schmidt Clinic, Wiesbaden, Germany; 11 Nephrology and Hypertension, University Medical Center Utrecht, Utrecht, Netherlands; 12 Department of Diagnostic and Interventional Radiology, Hannover Medical School, Hannover, Germany; 13 Radiologie München, Munich, Germany; 14 European Cyberknife Center Munich-Großhadern, Munich, Germany; 15 Department of Urology, Ludwig-Maximilians University Munich, Munich, Germany

**Keywords:** Renal cancer, Bone metastases, Cancer therapy, Cancer imaging

## Abstract

Bone is a major site of haematogenous tumour cell spread in renal cell carcinoma (RCC), and most patients with RCC will develop painful and functionally disabling bone metastases at advanced disease stages. The prognosis of these patients is generally poor and the treatment is, therefore, aimed at palliation. However, RCC-associated bone metastases can be curable in select patients. Current data support a multimodal management strategy that includes wide resection of lesions, radiotherapy, systemic therapy, and other local treatment options, which can improve quality of life and survival. Nevertheless, the optimal approach for metastatic bone disease in RCC has not yet been defined and practical recommendations are rare. To improve the management and outcomes of patients with RCC and bone metastases, the International Kidney Cancer Coalition and the interdisciplinary working group on renal tumours of the German Cancer Society convened a meeting of experts with a global perspective to perform an unstructured review and elaborate on current treatment strategies on the basis of published data and expertise. The panel formulated recommendations for the diagnosis and treatment of patients with RCC and metastasis to the bone. Furthermore, the experts summarized current challenges and unmet patient needs that should be addressed in the future.

## Abstract (web summary)

In this Expert Consensus, Grünwald et al. summarize their recommendations for the diagnosis and treatment of patients with renal cell carcinoma and metastasis to the bone. They also outline current challenges and unmet patient needs that should be addressed in the future.

## Introduction

Haematogenous spread to the bone is common in patients with renal cell carcinoma (RCC)^1^. Around one-third of patients with metastatic RCC are diagnosed when they already have bone metastases and another third of patients will develop them throughout their future disease course^2^. Before the introduction of targeted therapy to clinical practice in RCC, the rate of skeletal-related events (SREs), defined as pathological fractures, radiotherapy, surgery, neural compression, or hypercalcaemia, was 74–85%^3^. Since the use of targeted antiangiogenic therapy in patients with advanced disease and the resulting extended overall survival, bone metastases have become even more prevalent^2–5^. Owing to their predominantly osteolytic nature, bone metastases in RCC can be associated with severe morbidity and predispose patients to skeletal complications^1–4^. Local treatments include radiotherapy in ~80% of patients and orthopaedic surgery in ~30% of patients^6^. However, pain is often poorly managed, indicating a need for improved treatment strategies^1,7,8^. Importantly, some patients are candidates for curative treatment. A multidisciplinary management approach is warranted to yield optimal outcomes, as no common diagnostic and therapeutic standard currently exists and clinical guidelines provide limited information on how to manage bone metastases from RCC^2,9–12^. A review of local therapies for RCC metastases in general revealed the poor evidence base for the treatment of bone metastases^13^.

In this Expert Consensus Document, we summarize the consented recommendations for the diagnosis and treatment of patients with RCC and metastasis to the bone. Furthermore, we outline current challenges and unmet patient needs that should be addressed in the future.

## Methods

The International Kidney Cancer Coalition (IKCC) — an independent global network of patient organizations with a focus on kidney cancer — and the interdisciplinary working group on renal tumours of the German Cancer Society met in Berlin, Germany, to elaborate recommendations on the basis of the available literature and experience. Invitees included a multidisciplinary panel of clinical experts from various institutions with ample experience in the treatment of patients with kidney cancer. The objective of the meeting was to identify unmet patient needs and to provide guidance for diagnosis and treatment of bone metastases from RCC in routine practice according to the literature, enhanced by expert opinion after thorough discussion. The different sections of this Expert Consensus Document, which describe the reported data, are followed by summaries of the available evidence, in which each point has been classified according to the level of evidence (LoE) of the available data (Table 1). Specific recommendations according to the panel’s position are then listed, and outstanding issues and patient needs are summarized.

**Table 1:** Levels of evidence

Level of evidence	Description
1	Large RCT with low potential for bias; meta-analyses of well-conducted RCTs
2	Other randomized trials or meta-analyses of such trials or of RCTs with demonstrated heterogeneity
3	Prospective cohort studies
4	Retrospective cohort studies or case–control studies
5	Studies without control group, case reports, experts opinions

This table is adapted from a table in the European Society for Medical Oncology (ESMO) Clinical Practice Guidelines Standard Operating Procedures^108^ that is used for evidence grading for the ESMO Clinical Practice Guidelines. RCT, randomized controlled trial.

## Epidemiology of RCC bone metastases

**Fig. 1:**
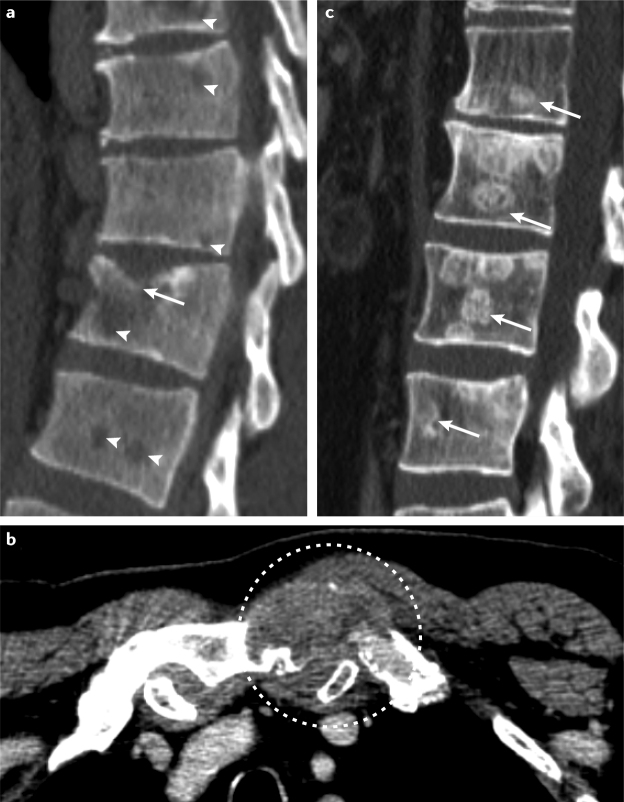
Radiographic pattern of RCC bone metastases. Bone metastases from renal cell carcinoma (RCC) can present in different patterns. **a** | Osteolytic bone metastases (arrowheads) that present with a symptomatic pathological fracture of the vertebral body (arrow) can be seen. **b** | Osteolytic bone metastases that present with an extraosseous soft-tissue portion in proximity to the sternoclavicular joint (dashed circle) can be seen. **c** | Bone metastases that result in bone formation (osteoblastic lesions) are rare (arrows).

Lung lesions are the most frequent manifestation of metastases in patients with RCC^14^. The bones are the second most common site for metastases from RCC^4,8,9,14,15^, and occurrence of bone metastases has been reported in 35–40% of patients with advanced RCC^9,12,16,17^. These metastases are predominantly found in the pelvis, sacrum, spine, and proximal extremities^18^ and are predominantly osteolytic lesions (79% osteolytic, 7% osteoblastic, and 13% mixed lesions)^15^ (Fig. 1). A study that included data from >1,800 patients found that 31% of patients had bone metastases at the time of RCC diagnosis, whereas 68% developed bone metastasis at a median period of 25 months throughout the course of their disease^15^. Most patients presented with multiple bone metastases (71%).

The median overall survival after diagnosis of RCC bone metastases ranges from 12 months to 28 months^19,20^. A retrospective study from 2016 analysed the association between the onset of bone metastases and outcome in 82 patients with RCC and bone metastases treated with sunitinib^21^. The time to clinical progression on sunitinib treatment was similar for patients with synchronous and metachronous bone lesions, but overall survival was longer for patients with metachronous bone lesions than for those with synchronous lesions (38.5 months (95% CI 15–62) versus 21.1 months (95% CI 16–26.2); *P* = 0.001), indicating a differential role of immediate and subsequent occurrence of bone metastases in RCC. The prognostic role of the number of bone metastases from RCC has been retrospectively analysed in 300 patients^22^. In this work, the number of bone metastases was associated with overall survival. Patients with 1, 2–5, or >5 bone metastases had a median overall survival of 28 months, 18 months, or 9 months, respectively. Patients with a solitary synchronous bone metastasis had the longest survival (40 months), a finding that is supported by other studies^23,24^. A long interval (≥24 months) from diagnosis of RCC to onset of bone metastases, having a solitary bone lesion, and the absence of extraosseous metastases are associated with prolonged survival^20,23^. A retrospective analysis of data from 94 patients suggested five risk factors to predict prognosis of patients with RCC and bone metastases: sarcomatoid differentiation of the primary tumour (*P* = 0.001), spinal involvement (*P* = 0.003), extraosseous metastasis (*P* = 0.021), increased alkaline phosphatase levels (>1.5 times the upper limit of normal; *P* = 0.0003), and increased C-reactive protein levels (>0.3 mg/dl; *P* = 0.018)^25^.

## Diagnostic evaluation of RCC bone metastases

Early diagnosis of bone metastases is essential for reducing morbidity and improving outcome^2,26^, for which appropriate imaging is needed. However, current imaging techniques are limited by their sensitivity (bone scintigraphy) and availability or costs (whole-body MRI). Current guidelines suggest imaging in the presence of symptoms only, for example, bone pain or fracture^11^, thus, restricting diagnosis to patients with debilitating or complicating metastases. In the absence of appropriate techniques, adequate follow-up monitoring with clinical surveillance is important in the management of patients with RCC. Of note, contemporary surveillance measures consist of routine images of the thorax and abdomen, which do not capture extremities and the neck, which needs to be considered in clinical decision-making. In the presence and/or persistence of clinical symptoms suggestive of bone disease, adequate imaging should be initiated.

### Imaging techniques in bone metastases

**Fig. 2:**
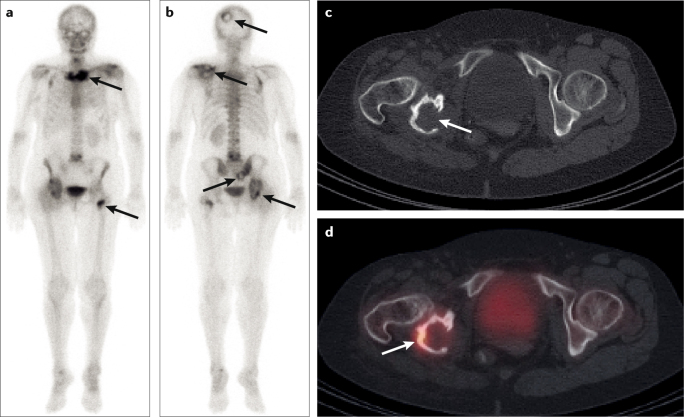
Radionuclide pattern of bone metastases from RCC. Multiple osseous bone metastases (arrows) detected by technetium-99 m (^99m^Tc)-phosphonate bone scintigraphy in anterior (part **a**) and posterior (part **b**) views. Transversal CT (part **c**) depicts osteolysis of the posterior right acetabulum (arrow), which shows only partially activated bone metabolism (arrow) on single-photon emission CT (SPECT)–CT (part **d**). RCC, renal cell carcinoma.

Technetium-99 m (^99m^Tc)-diphosphonate bone scintigraphy, which provides assessment of the whole skeleton, and conventional radiography are widely used for the assessment of bone metastases^2^ (Fig. 2). In most instances, only loss of bone mineral content ≥50% can be detected on radiographs, which limits their utility for detection of RCC metastases to the bone^2,27^. The osteolytic nature of RCC bone metastases decreases sensitivity of bone scans to ≤50%, as bone scans depict only the osteoblastic reaction of bone to metastatic tissue, which is often absent in RCC lesions^28^. The deposition of bone-seeking radiopharmaceuticals occurs through physicochemical adsorption (chemisorption) to the hydroxyapatite structure of bone tissue. Purely osteolytic lesions in which bone structure is displaced by tumour cells cannot be depicted by these radiopharmaceuticals; thus, false-negative findings are common for osteolytic lesions.

**Fig. 3:**
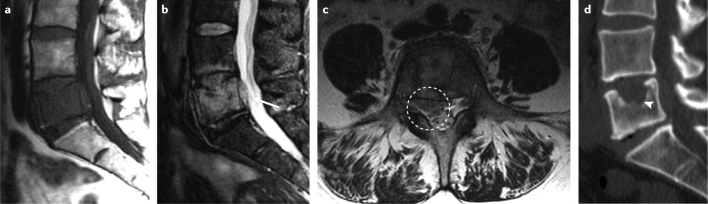
Imaging techniques for RCC bone metastases. Bone metastases of the lumbar spine are detected by MRI or CT. T1-weighted sagittal (part **a**), T2-weighted sagittal (part **b**) and T2-weighted axial (part **c**) MRIs show the destruction of the fifth lumbar vertebra. MRI offers benefits for the assessment of the spinal canal and its possible involvement (arrow and dashed circle). By contrast, CT imaging (part **d**) has the advantage of displaying the structure of the mineral bone and enables assessment of its stability in the case of osteolysis (arrowhead). RCC, renal cell carcinoma.

CT is an important tool for the assessment of bone stability and structure^27^ (Fig. 3). Given the pattern of metastatic spread to the proximal axial skeleton, which is covered by CT of the chest, abdomen, and pelvis, most lesions that cause substantial bone loss are captured by CT; hence, follow-up monitoring can be based on routine CT imaging that includes the proximal extremities and sagittal reformations of the spine using bone windowing and a bone-specific reconstruction technique.

The true value of PET–CT with ^18^F-sodium fluoride (^18^F-NaF) or ^18^F-fluorodeoxyglucose (^18^F-FDG) in staging RCC remains to be determined. ^18^F-NaF-PET–CT seems to have a higher sensitivity and accuracy than bone scan or CT in the detection of RCC bone metastases, but data are limited owing to small patient cohorts and a low number of comparative studies^29^. ^18^F-FDG-PET without CT has demonstrated high specificity but limited sensitivity for detection of distant RCC metastases in older studies before the introduction of hybrid PET–CT, providing an overall specificity and sensitivity of 100% and 63.6%, respectively^30^. Hybrid ^18^F-FDG-PET–CT combines the advantages of both PET and CT and has shown high sensitivity for the detection of disease recurrence or metastasis in the post-operative surveillance of patients with advanced RCC (89.5% sensitivity and 83.3% specificity)^31^. ^18^F-NaF-PET–CT has demonstrated even higher sensitivity, albeit in small patient cohorts^29^. However, PET–CT is not routinely used, owing to high associated costs and availability. This technique can be used as an adjunct when conventional imaging is not conclusive, as early diagnosis of metastatic disease can drastically alter the therapeutic management.

According to the literature, MRI has >93% sensitivity and specificity for the detection of skeletal metastases^2,28^ (Fig. 3). MRI is not routinely used for patient follow-up monitoring, owing to high associated costs and limited availability. In patients with seemingly solitary or oligometastatic spread to the bone, whole-body MRI should be performed before extensive surgical resections to ensure that the patient truly has a limited number of bone lesions amenable to surgical management.

### Contrast agents and renal impairment

A systematic review and meta-analysis assessed the risk of acute kidney injury (AKI) from intravenous contrast medium in patients undergoing CT imaging with and without intravenous contrast agent for various indications, including metastatic RCC^32^. The risks of AKI, death, and dialysis were similar for both groups (relative risk (RR) 0.79, 95% CI 0.62–1.02; RR 0.95, 95% CI 0.55–1.67; and RR 0.88, 95% CI 0.23–3.43, respectively). This pattern was observed regardless of contrast medium type or whether patients had renal insufficiency^32^, indicating that patients with RCC do not have an increased risk of AKI from intravenous contrast medium. Individual factors other than the use of contrast medium are more likely to contribute to the development of AKI. In detail, patients at low risk (serum creatinine level (SCr) ≤1.5 mg/dl) and intermediate risk (SCr 1.6–2.0 mg/dl) can be safely scanned with intravenous contrast medium, as they do not have a substantial risk of AKI. Generally, CT and MRI scans should be acquired with intravenous contrast medium unless patients have substantial impairment of renal function (SCr >2.0 mg/dl).

### Summary of evidence

- Bone scintigraphy has low sensitivity to detect bone metastases (LoE: 3).
- CT imaging is a powerful tool to assess bone structure for detection of bone metastases, with a higher sensitivity than bone scintigraphy (LoE: 4).
- MRI has very high sensitivity and specificity to detect bone metastases (LoE: 4).
- PET has high specificity, and its sensitivity depends on the applied radiotracer (LoE: 3).
- Individual parameters rather than use of contrast medium are associated with AKI (LoE: 4).

### Panel’s position and recommendations

- CT of the chest, abdomen, and pelvis, including the proximal extremities and a sagittal reconstruction of the spine using a sharp bone reconstruction kernel, is standard for diagnosis and follow-up monitoring. Biphasic contrast agents are required to characterize visceral lesions.
- In asymptomatic patients, we do not recommend specific screening for bone metastases apart from routine tumour staging by CT of the thorax, abdomen, and pelvis.
- Bone scintigraphy and 18F-FDG-PET–CT should not be routinely used to screen for bone metastases in patients with RCC.
- Conventional radiography should be considered as an initial diagnostic procedure only in patients with symptomatic bone metastases.
- MRI should be used as an extended diagnostic procedure before local treatment in suspected skeletal-oligometastatic disease, as this technique has very high sensitivity in the detection of bone metastases, and its sensitivity exceeds that of bone scintigraphy.
- MRI is the preferred imaging modality to assess the extent of epidural disease in spinal metastases.
- PET can be used as an adjunct procedure when conventional imaging is not conclusive.
- We recommend biopsy for nonresectable bone metastases in the absence of prior pathological diagnosis of metastatic disease.

### Outstanding issues (unmet patient needs)

- The optimal screening method for patients with a solitary bone lesion still needs to be determined.
- The availability of whole-body MRI needs to be improved, as this technique is currently the best bone screening method.
- The comparative performance of whole-body MRI and PET for detection of bone metastases needs to be determined.
- A more precise surveillance algorithm for monitoring patients with bone metastases from RCC is needed.

## Local therapies for RCC bone metastases

Palliative treatment for patients with multiple bone metastases can include surgery (including minimally invasive methods, such as osteoplasty), radiotherapy, and pharmacological (including analgesic) therapy^9,33,34^. Solitary or oligometastatic disease should be treated with curative intent, preferably with wide resection margins.

### Surgery

Indications for surgical intervention in patients with metastatic RCC to the bone are intractable pain, presence of or impending pathological fracture, spinal instability, spinal cord compression, or curative intention^2,9,12^. Treatment goals are improvement of prognosis, local tumour control, pain relief, and preservation or reconstitution of function. Surgical procedures include resections with or without reconstruction, internal fixation, and neural decompression^2^. The primary tumour and the metastasis can be resected during the same operation or at different times^12^.

Surgical resection of solitary or oligometastatic lesions can improve the prognosis of patients with bone metastases, supporting a multidisciplinary team approach for treatment planning in these patients^11,24,35–37^. In patients with a favourable or intermediate prognosis, tumour-free margins were associated with favourable survival and local tumour control; having a wide surgical margin compared with an intralesional margin improved 5-year overall survival significantly from 11% to 31% (*P* = 0.028)^24^. Thus, wide resection of lesions should be favoured. The effect on survival also applies to patients with a limited amount of resectable osseous and visceral metastases. If all metastatic lesions can be resected, 5-year overall survival increases from <10% to >40%^24^. Poor prognosis is associated with multiple nonresectable skeletal metastases, concomitant visceral metastases, and local recurrence^38^. Nevertheless, these factors should not exclude patients from receiving more elaborate reconstructions, such as endoprosthetic replacement or bone transplantation for functional improvement, as survival >1 year can still be expected in many patients^24,38^. An alternative approach for patients with spinal metastases involving epidural disease can involve multimodal therapy, consisting of surgery followed by definitive spine radiosurgery^39^. If rapid spinal decompression is required, direct decompression surgery plus radiotherapy was superior to radiotherapy alone in a randomized trial in 101 patients (OR 6.2, 95% CI 2.0–19.8; *P* = 0.001)^40^.

In one study including 45 patients with a total of 56 lesions, surgery was associated with pain relief (91% of patients), good-to-excellent functional outcome (89%), and local tumour control (local recurrence in 7.1% of patients)^36^. Randomized studies that compare different treatment modalities are lacking, but surgery is recommended if a curative resection is intended and metastases are completely resectable, according to the German guideline on RCC^9^.

### Summary of evidence

- Complete tumour resection is associated with locoregional control and survival (LoE: 5).
- Long-term survival can be achieved in patients with solitary bone metastasis (LoE: 5).
- If spinal cord decompression is required, surgical resection plus radiotherapy is superior to radiotherapy alone (LoE: 2).

### Radiotherapy

Radiotherapy has important functions in the palliative management of bone lesions, including pain control, relief of spinal cord compression, and support of bone remineralization^41^. Historically, RCC bone metastases were considered resistant to conventional fractionated radiotherapy^42^. In the past 5 years, hypofractionated regimens, consisting of a single or few fractions, have been established and are considered to be effective in metastatic RCC^43^. Stereotactic body radiation therapy (SBRT) delivers high doses confined to the tumour^43–45^ and has been reported to be feasible in patients with oligometastatic disease (local control rate >90%)^42,45–47^. In animal models, single-dose SBRT has been associated with vascular damage^42,43^, suggesting synergistic effects with targeted agents in RCC^45^. An optimal schedule for combined treatment in humans has not yet been determined^48^. Phase I clinical trials have tested sunitinib or pazopanib in combination with radiotherapy in patients with RCC or central nervous system (CNS) malignancies^49,50^. In patients with metastatic RCC, the recommended dose for SBRT in combination with 800 mg pazopanib per day was 36 Gy in 3 fractions^49^. Another phase I trial tested daily 37.5 mg sunitinib in combination with various radiotherapy doses and fractions for different CNS malignancies^50^. Keeping in mind the variety in patients and radiotherapy regimens, this study reported acceptable toxicities, supporting the combined use of radiotherapy and sunitinib.

Data for SBRT for metastatic RCC mainly come from small series using different doses and schedules. Patients with various metastatic sites and locations of the primary tumour have been treated with SBRT. SBRT resulted in complete regression of irradiated lesions in 30% of patients and tumour shrinkage or stabilization in an additional 60% of patients, indicating high local control rates in 90–98% of patients as well as pain control; treatment related adverse events occurred in 39.7% of patients^51^. A randomized trial in patients with bone metastases that included 41 patients with RCC showed that the use of 8 Gy in a single fraction was not inferior to and was less toxic than 20 Gy given in multiple fractions^52^. 30 Gy in 10 fractions is a commonly used regimen for treatment of bone metastases and has also been shown to reduce pain from bone metastases in patients with RCC^53^. In a retrospective study, SBRT rapidly improved symptoms and resulted in more durable clinical and radiographical responses than conventionally fractionated external beam radiotherapy in patients with metastatic RCC to the bone^42^. No uniform dose and fractionation was used in this study, but a biologically effective dose of ≥80 Gy was associated with improved clinical local control (HR 0.140, 95% CI 0.025–0.787; *P* = 0.26)^42^. In another study, single-dose SBRT (24 Gy) compared with hypofractionated SBRT resulted in improved 3-year local progression-free survival (88% versus 17%; *P* < 0.001)^54^. In this study, only one patient had a treatment-induced vertebral fracture^54^, but another report has reported fractures in 7.2% of patients following single-fraction 24 Gy SBRT, indicating a potential severe late adverse event of spinal SBRT^55^.

The incidence of vertebral compression fracture (VCF) was reported to be <5% for conventional radiotherapy^56^, but the incidence of VCF seems to be higher for spine SBRT, which was reported to occur in 11–18% of lesions^45,57–61^. Hence, salvage treatment is required in some patients. One study reported that up to 47% of lesions required salvage kyphoplasty, surgery, or both^58,61^. The greatest risk factors for VCF were reported to be a high dose per fraction (≥20 Gy), osteolytic disease, and pre-existing VCF or spinal deformity^58,59^.

Overall, clinical results showed that single-session and multiple-session stereotactic spinal radiosurgeries are noninvasive, safe, and effective treatments for patients with spinal lesions. A single fraction provides more rapid symptom and quality of life improvement but is associated with a higher re-treatment rate in long-term survivors (1% versus 13%, *P* < 0.001)^41,62^.

### Thermal ablation

Radiofrequency ablation is a percutaneous method for symptom relief and local control of bone metastases^63,64^, which can be combined with vertebroplasty to stabilize vertebrae^65,66^. Radiofrequency ablation can destroy the metastatic tumour tissue but leaves a cavity in the bone, which reduces its mechanical stability^66^. Ex vivo testing in a human spine model showed that the combination of radiofrequency ablation and vertebroplasty resulted in improved stability and axial loading of the vertebrae compared with ablation alone^66^, highlighting the importance of multimodal concepts in the treatment of bone metastases in metastatic RCC. Percutaneous image-guided ablation has been advocated to relieve pain, reduce tumour burden, and provide mechanical stabilization of the bone^67^. Initial results suggest that radiofrequency ablation in patients with painful bone metastases is safe and reduces pain levels (efficacy in 23 of 33 patients (69.7%))^68^. The analgesic effect is reported to reach significance as early as day 7 after radiofrequency ablation, highlighting the early onset of clinical efficacy of this technique^69^. The applicability of radiofrequency ablation is limited by the cooling effect of local blood flow (heat sink effect) and the high electrical impedance of tissues. A study from 2018 compared the heat sink effect for radiofrequency and microwave ablation in the liver. Microwave ablation maintained a consistent surface temperature irrespective of the proximity of vessels, whereas a temperature drop by one-third near vessels was noted for radiofrequency ablation, indicating the limitations of this technique^70^.

The efficacy of cryoablation for the treatment of RCC was first described in the early 1970s^71^. Cryoablation is currently used to treat either RCC primary tumours or metastases, including lesions of the bone, and is an alternative to radiofrequency ablation^72,73^. In a retrospective series, cryoablation of bone metastases from RCC resulted in high local tumour control in 81% of 50 lesions^74^. Preliminary data indicate similar levels of pain relief (68% and 64% of patients for cryoablation and radiofrequency ablation, respectively) and a significant increase in quality of life (*P* < 0.001) for both approaches^75^. Future work should investigate differences between these techniques to support appropriate use of the most adequate technique in the individual patient.

### Summary of evidence

- Tyrosine kinase inhibitors (TKIs) can be given concomitantly with radiotherapy (LoE: 3).
- Hypofractionated radiotherapy is superior to standard fractionated radiotherapy (LoE: 4).
- SBRT has promising clinical effectiveness (LoE: 3).
- Radiotherapy should be performed after decompressing surgery for metastases of the spine (LoE: 2).
- Radiofrequency ablation exerts local tumour control and reduction of symptoms (LoE: 5).
- Cryoablation might be used to control the primary tumour or bone metastases (LoE: 5).

### Panel’s position and recommendations

- Adequate pain therapy is the mainstay in the management of patients with symptomatic bone metastases.
- Local therapies are the preferred choice of treatment to achieve tumour control.
- We recommend giving priority to local treatment of bone metastases in the case of instability, fracture, pain, or neurological impairment or as an individual decision based on a patient’s risk profile.
- Wide resection with curative intent is the primary approach to solitary or oligometastatic bone metastasis. Even in patients with further metastatic disease, local wide resection might substantially and durably improve quality of life; however, mutilating surgery should be avoided.
- Additive radiotherapy after resection of bone metastases resulting in negative margins (locoregional R0) is not indicated.
- Medical treatment after resection resulting in negative margins (locoregional R0) is not indicated; however, in the presence of residual disease or additional metastases and lack of local therapeutic options, medical treatment should be offered.
- Radiotherapy should be given as a single high-dose (radiosurgery) or hypofractionated stereotactic regimen.
- We do not recommend conventionally fractionated radiotherapy for the treatment of RCC.
- In patients who require both medical treatment and radiotherapy, we suggest combining both treatment modalities.
- Symptomatic bone metastases should preferably receive wide resections whenever feasible or, otherwise, intralesional resections and reconstructions or SBRT in inoperable cases.
- Radiofrequency ablation, microwave ablation, and cryoablation are treatment options in individual patients with bone metastases <3 cm.
- Patients with asymptomatic bone metastases should receive active surveillance, pre-emptive local therapy (in high-risk patients), or systemic therapy, if appropriate.

## Medical therapy

### Targeted therapies

TKIs and anti-vascular endothelial growth factor (VEGF) antibodies are widely used as first-line and second-line treatments of advanced RCC^12^. Direct evidence on the effects of targeted agents on bone metastases is currently limited to a few studies, which suggest that TKIs can extend the mean time to progression of existing bone lesions and reduce formation of new bone lesions^2,76^.

A retrospective analysis of data from 375 patients with metastatic RCC showed that patients with bone metastases treated with the first-generation TKIs sunitinib or sorafenib had improved overall survival compared with historical controls (24 months versus 18 months; *P* < 0.01)^77^. This finding is supported by another retrospective study, which compared the effect of IFNα, sunitinib, and sorafenib on the occurrence and progression of metastatic bone lesions in 292 patients with RCC^76^. Sunitinib decreased the formation (*P* = 0.034) and time to new bone lesions (*P* = 0.047) compared with sorafenib. Preclinical evidence suggests that sunitinib inhibits osteoclast activity, corroborating this clinical observation^78^.

Treatment with cabozantinib, a third-generation TKI, resulted in improved survival in two clinical studies, indicating the important role of this class of agents in the treatment of these patients^79^. The subgroup analyses of patients with bone metastases in the phase II first-line study comparing cabozantinib and sunitinib and the second-line phase III study comparing cabozantinib and everolimus found improved progression-free survival with cabozantinib treatment (HR 0.54, 95% CI 0.31–0.95 and HR 0.33, 95% CI 0.21–0.51, respectively)^79^. In second-line use, cabozantinib was also associated with a better overall survival (HR 0.54, 95% CI 0.34–0.84). Results of an in vitro study suggest that these effects are mediated by direct inhibition of osteoclast activity and modulation of the bone microenvironment through alteration of the receptor activator of nuclear factor-κB ligand (RANKL; also known as TNFSF11):osteoprotegerin ratio in osteoblasts^80^. In addition, treatment with nivolumab, an antibody against programmed cell death protein 1 (PD1), resulted in improved survival compared with everolimus in patients with metastatic RCC, which was maintained in the subgroup of patients with bone metastases^81^.

### Bisphosphonates and antibodies

Bone-targeting agents such as bisphosphonates and denosumab have been shown to reduce SREs and the worsening of pain associated with bone metastases and to improve the control of hypercalcaemia in patients with different advanced malignancies, including RCC^82,83^. Thus, bone-targeting agents are recommended for patients with or without pain caused by bone metastases from solid tumours^84^. Current evidence supports the surgical and/or radiotherapeutic treatment of bone metastases in patients with RCC, but the role of systemic bone-targeted treatment is less well defined.

#### Bisphosphonates

Bisphosphonates are a heterogeneous group of bone-targeting agents, which are absorbed by active osteoclasts, thereby blocking their activity^85^. Zoledronic acid is a potent inhibitor of osteoclast activity and is commonly used for treatment of bone metastases in patients with RCC^26^. Data in patients with RCC and bone metastases are limited to a subgroup analysis from a pivotal phase III study (*n* = 74) and a phase II trial (*n* = 50), which were performed when targeted therapies for RCC were not yet available^86–88^. In these trials, zoledronic acid treatment decreased the rate of SREs to 22–37%, which compared favourably with a rate of 74% SREs in the placebo control arm of the phase III study^86–88^. By contrast, a pooled analysis of data from 2,794 patients with RCC mainly treated with targeted therapies did not show a correlation between bisphosphonate, including zoledronic acid, use and outcomes (progression-free survival, overall survival, or SRE incidence)^89^. Interestingly, the rates of SREs in patients with or without bisphosphonate use were 8.6% and 5.8% (*P* = 0.1785), respectively, possibly underscoring the value of active antitumour therapy given to patients with RCC in recent years. Furthermore, an increase in hypocalcaemia, renal insufficiency, and osteonecrosis of the jaw (ONJ) was associated with the use of bisphosphonates (*P* < 0.0001)^89^. Adverse effects can be substantial. Atypical femur fractures have been reported for long-term use of bisphosphonates, including zoledronic acid^90^. In addition, zoledronic acid requires renal function monitoring and corresponding dose adjustments^44^, which is of particular relevance in patients with RCC^2^ and are not required for denosumab treatment. As a class effect, bisphosphonates are associated with the risk of hypocalcaemia and, therefore, require calcium and vitamin D supplementation. Overall, bisphosphonates alone are of limited value in patients with bone metastases from RCC and are often used as an adjunct to targeted therapies. While the value of bisphosphonates in combination with TKIs remains unclear, such combinations have been associated with an increase of the incidence of ONJ^89,91^.

#### Denosumab

Denosumab inhibits RANKL, thereby blocking osteoclast activity^2,12,26,44^. A phase III trial demonstrated noninferiority for denosumab compared with zoledronic acid in delaying time to first on-study SRE in patients with bone metastases from various primary cancers (HR 0.84, 95% CI 0.71–0.98)^83^. In the subgroup of patients with solid primary tumours, denosumab was more effective in delaying or preventing SREs in patients with bone metastases (HR 0.81, 95% CI 0.68–0.96) and also prevented pain progression (HR 0.83, 95% CI 0.71–0.97) compared with zoledronic acid^92^. In a meta-analysis of data from 3 pivotal trials in >5,700 patients, denosumab improved time to first SRE by a median of 8.21 months and reduced the risk of a first SRE by 17% compared with zoledronic acid^93^. The pivotal phase III trial included patients with various cancers and bone metastases, but no specific subgroup analyses for those with RCC was reported^83^, precluding a recommendation of denosumab in patients with metastatic RCC.

#### Osteonecrosis of the jaw

ONJ is a rare (1.3–1.8%) but potentially serious event in patients with cancer who receive bisphosphonates or denosumab^2,44,94^. Some evidence exists that the combination of bisphosphonates and antiangiogenic therapies is more frequently associated with ONJ than bisphosphonates alone^89,91^. Published consensus recommendations^95^ and evidence-based guidelines for the prevention and management of ONJ include regular dental examinations before and during treatment, elimination or stabilization of oral disease before initiation of these agents, and maintenance of good oral hygiene^9^.

### Combination therapy

Zoledronic acid treatment has been associated with response to radiotherapy in six of ten patients with bone metastases from RCC^96^ and with a favourable SRE-free survival and pain response compared with radiotherapy alone^97^, possibly mediated by a radiotherapy-sensitizing effect of zoledronic acid on RCC cells^98^. Simultaneous antiangiogenic therapy and stereotactic radiosurgery in patients with spinal or cerebral metastases from RCC was associated with high local tumour control (98% of patients) after a 15-month follow-up period without an increase in adverse events, indicating that combination therapy can be offered to select patients^99^.

### Summary of evidence

- TKIs have clinical activity in metastatic RCC with bone metastases (LoE: 2).
- Cabozantinib treatment resulted in superior outcomes compared with everolimus or sunitinib in patients with metastatic RCC and bone metastases (LoE: 2).
- Bisphosphonates decrease SREs in patients with metastatic RCC and bone metastases (LoE: 2).

### Panel’s position and recommendations

- Pharmacological treatment for bone metastases is given with palliative intent only.
- Targeted therapies should be used to treat bone metastases that are not amenable to local therapies.
- Among available targeted therapies, cabozantinib should be used preferentially in patients with multiple bone metastases.
- Bone-targeted agents should be used to control tumour-associated hypercalcaemia.
- Whether bisphosphonates or denosumab treatment in addition to targeted therapy improves clinical outcomes is uncertain.
- The combination of osteoprotective therapy and targeted agents increases the risk of adverse events, such as ONJ.
- No reliable evidence of a long-term osteoprotective treatment exists; hence, the duration of therapy should be chosen on the basis of disease stage, individual risk, and symptoms.
- If bone-directed symptoms recur during pauses of anticancer drug treatment, an alternative dose regimen should be considered (continuous therapy or shortening of the drug-free interval).
- Early, immediate, and individual pain therapy should be offered to symptomatic patients and requires continuous monitoring. Local therapy should be considered again if symptoms worsen.

### Outstanding issues (unmet patient needs)

- The benefit and duration of osteoprotective therapy in patients with RCC and bone metastases who receive targeted therapies remain to be defined.
- A clinical need exists in the palliative setting to improve systemic therapies to control bone metastases effectively in terms of response and symptoms.

## Other therapies

### Radionuclide therapy

Radionuclide therapy of bone metastases with bone-seeking radiopharmaceuticals, such as samarium-153-ethylene diamine tetra(methylene phosphonic acid) (^153^Sm–EDTMP)^100–103^, is not indicated outside of clinical studies owing to the predominantly osteolytic nature of bone metastases in RCC. The deposition of bone-seeking radiopharmaceuticals occurs at the mineralization front of bone (osteoid) but not near osteoclasts and not within the osteolytic metastatic lesion itself that has displaced the normal hydroxyapatite structure of bone tissue.

### Therapeutic embolization

Transarterial embolization is generally used in primary or metastatic bone tumours^63^ to reduce operative haemorrhagic risks or to simplify or enable more definitive surgery or in the context of palliation^104^. Clinical response to embolization has been reported in patients with RCC^105–107^. A small series including 21 patients and 39 metastatic bone lesions reported a clinical response in 36 lesions, with a median response duration of 5.5 months^105^, highlighting a role of embolization in select patients.

### Panel’s position and recommendations

- Radionuclide therapy should not be used outside of clinical studies owing to limited established data.
- Transarterial embolization should be considered before resection of bone metastases.

## Conclusions

**Fig. 4:**
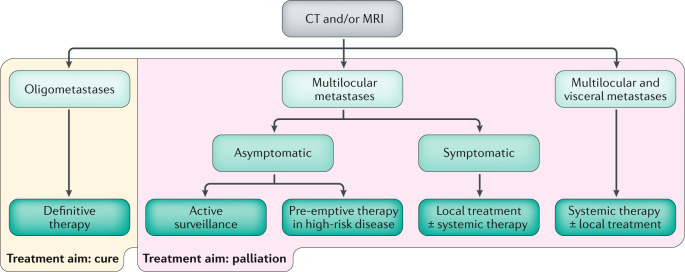
Proposed treatment algorithm for patients with RCC and bone metastasis. The multidisciplinary expert panel proposes an algorithm for the management of patients with metastatic bone disease arising from renal cell carcinoma (RCC). The extent and location of metastasis should be assessed using CT and/or MRI. In patients with oligometastatic bone disease, disease cure is the aim of treatment and surgery is the preferred treatment option. However, other definitive therapy options might also be applicable, and the approach should be individualized to the needs of the patient. In other patients, management aims to palliate symptoms. In patients with multilocular bone metastases, treatment choice depends on the presence of symptoms. Asymptomatic patients can either undergo active surveillance or pre-emptive therapy in cases of high-risk disease. Symptomatic patients with multilocular disease should be assessed for local treatment first. Instability, fracture, pain, neurological impairment, and individual decision should be used for proper clinical judgement of local therapies. Surgery with or without radiotherapy remains the mainstay of treatment in symptomatic disease, but the approach should be individualized. Medical treatment can be given in the presence of residual disease or additional metastases but is not recommended as an adjunct after complete resection or definite locoregional treatment. In patients with multilocular bone and visceral metastases, systemic therapy is the mainstay of treatment, which can be amended by local treatments depending on pain, fracture, instability, or neurological symptoms. Bone-targeted agents can be used in patients with multilocular bone disease as an adjunct to locoregional or systemic therapies, which are the cornerstones to treat bone disease from RCC. An individual decision should be made for the duration of bone-targeted therapies, as specific adverse events of the bone can occur with long-term use. Stage of disease, individual risk of local complications, and patient symptoms should be used for clinical decision-making.

On the basis of the available evidence and expert opinion, we propose an algorithm for the clinical management of patients with RCC and bone metastasis (Fig. 4). Multidisciplinary care is essential for maximizing patient benefit, and adequate pain management should be ensured in all patients. Surgery is a curative approach in select patients. If indicated, surgery should be performed before commencing pharmacological treatment. Considerable technological advances in radiotherapy, such as SBRT and stereotactic radiosurgery, have enabled the delivery of high doses with an accuracy within millimetres, which broadens our perception of the use of radiotherapy beyond the scope of symptom control. We propose that medical drug treatment should not be discontinued when radiotherapy is given. Bone metastases respond well to systemic therapy, and an added value has been described for systemic targeted therapy. However, the most active agent for the treatment of bone metastases still needs to be defined.

Data on the use of osteoprotective treatment in patients with RCC remain unsatisfying. Targeted therapies boosted the effectiveness of pharmacological treatment, including responses in patients with bone metastases. Whether osteoprotective measures are necessary in the context of targeted therapies remains unclear, as the largest retrospective series does not support this notion. However, the combination of osteoprotective and targeted therapy comes at the expense of additional and sometimes debilitating toxicities. Personalized therapy for patients with RCC and bone metastases remains an important topic and offers several clinical questions for future research. The main goal is to incorporate patients’ needs into the management strategy for bone metastases from RCC.
